# Oral pathology and oral medicine in Latin American countries: current stage

**DOI:** 10.4317/medoral.26500

**Published:** 2024-04-14

**Authors:** Éder Gerardo Santos-Leite, Layanne Sobral, Gerardo Gilligan, Janeth Liliam Flores-Ramos, Wilfredo Alejandro González-Arriagada, Claudia Pena Vega, Patricia Reiván-Ortiz, María del Carmen González-Galván, Wilson Delgado-Azañero, Ronell Bologna-Molina, Mariana Villarroel-Dorrego, Ricardo Martinez-Pedraza, Bruno Augusto Benevenuto de Andrade, Hercílio Martelli-Júnior

**Affiliations:** 1 Oral Diagnosis Department, Piracicaba Dental School, University of Campinas, Piracicaba, São Paulo, Brazil; 2 Department of Oral Diagnosis, Dental School, State University of Montes Claros, Montes Claros, Minas Gerais, Brazil; 3 Oral Medicine Department, Facultad de Odontología, Universidad Nacional de Córdoba, Argentina; 4 School of Dentistry, Universidad Mayor de San Andrés, La Paz, Bolivia; 5 Service of Oral and Maxillofacial Pathology, School of Dentistry, Universidad de los Andes, Santiago, Chile; 6 School of Dentistry, National University of Colombia, Colombia; 7 School of Dentistry, Universidad Viña del Mar, Chile; 8 Faculty of Dentistry, National University of Asunción, Paraguay; 9 Department of Oral Pathology, Oral Medicine and Oral and Maxillofacial Surgery, School of Dentistry, Universidad Peruana Cayetano Heredia, Lima, Perú; 10 Molecular Pathology Area, School of Dentistry, University of the Republic, Montevideo, Uruguay; 11 Universidad Central de Venezuela, Caracas Venezuela; 12 School of Dentistry, Universidad Autónoma de Nuevo León, Nuevo León, Mexico; 13 Department of Oral Diagnosis and Pathology, School of Dentistry, Universidade Federal do Rio de Janeiro; 13 Center for Rehabilitation of Craniofacial Anomalies, Dental School, University of José Rosario Vellano, Alfenas, Minas Gerais, Brazil

## Abstract

**Background:**

Oral Pathology (OP) and Oral Medicine (OM) are specialties in dentistry whose main objective is the diagnosis and treatment of oral and maxillofacial diseases, and aspects related to the academic training of professionals and fields of practice are distinct and heterogeneous around the world. This study aimed to evaluate professional training and areas of activity in OP and OM in Latin American countries.

**Material and Methods:**

A questionnaire was sent to 11 countries, with a professional in each country responsible for answering it. The questionnaire had 21 questions related to the process of professional training, areas of practice, the existence of scientific events in each country, and also collected demographic and population information.

**Results:**

OP and OM are practiced in all the countries studied, but the specialty is not recognized in all of them. Brazil was the first to recognize both as a specialty. Postgraduate programs designed to train specialists are available in various countries. Two countries offer residency programs, 6 countries provide specialization courses, 6 offer master's programs, and 3 have doctoral programs. Brazil boasts the highest number of undergraduate courses (*n*=412), while Uruguay has the lowest (*n*=2). Professional societies representing the specialty exist in ten countries. Brazil has the highest number of OP and OM specialists (*n*=422 and 1,072), while Paraguay has the smallest number (*n*=1 and 3).

**Conclusions:**

Although both specialties are widely practiced around the globe, professional training, the number of dentists trained and the fields of professional practice are very different between the countries studied.

** Key words:**Oral pathology, oral medicine, dentistry, education.

## Introduction

Oral and Maxillofacial Pathology (OP) and Oral Medicine (OM) are closely related areas of specialization and are considered vital to a comprehensive healthcare system (1). In Southern Europe and Ibero-American countries, such as Brazil, the term “Stomatology” is used to define the specialty of OM (2). The National Commission on Recognition of Dental Specialties and Certifying Boards of the United States defines OP as the dental specialty that deals with the nature, identification, and management of diseases affecting the oral and maxillofacial regions (https://ncrdscb.ada.org/).

OM is considered a young specialty of dentistry recognized across the world (2). It aims to diagnose and to provide (mostly non-surgical) treatment for primary diseases of oral mucosa and the jawbones, as well as salivary glands disorders, orofacial pain, and maxillofacial manifestations of systemic diseases and their treatment, such as cancer, infectious diseases, auto-immune disorders, and others. Furthermore, OM specialists provide comprehensive dental care for patients within a range of complex medical scenarios that impact oral health, including radiation therapy, chemotherapy, bone marrow transplantation, molecular targeted therapy in oncology, bone-modifying agents and antiresorptive drugs, cardiopathies, solid organ transplantation, AIDS, and COVID-19 (3,4).

During the COVID-19 pandemic, both areas of OP and OM had highlights, despite all the sanitary limitations observed. Discussions on teaching methodologies (5), forms of continuing education in the OP and OM (6), adequacy of the functioning of oral pathology laboratories (7), descriptions of oral lesions (8), evaluation of remote teaching in master programs (9), implication in oral oncology practice (10), were some of the topics studied.

Some studies around the world have analyzed the state of art and professional perspectives in OP and OM (2,3,11,12). However, no previous studies specifically evaluated the current stage of OP and OM in the context of South America and Mexico. Therefore, this study evaluated different dimensions of the two specialties, OP and OM, in the aforementioned countries.

## Material and Methods

A cross-sectional, observational and convenience study was conducted. A questionnaire was sent to ten countries in South America (Argentina, Bolivia, Brazil, Chile, Colombia, Ecuador, Paraguay, Peru, Uruguay and Venezuela) and Mexico. In each country, a professional responsible for collecting the requested data was chosen.

After the invitation of the OP and/or OM specialists, we sent a structured questionnaire, by personal e-mail, with 21 questions involving the specialties and characteristics of each country. The questionnaire included details of country demographics and the current status of the two specialties mentioned. Demographic data were consulted from population-based sources provided by official government bodies in the respective countries covered by the study. While data related to the specialty and training in dentistry in the country were collected from official government sources and the professional councils in each participating country in the study. Among the questions related to the specialties of OP and OM were: official recognition of OM and OM as a specialty in each country and the year of this event; the existence of a national scientific society; existence of scientific events in the country; existence of postgraduate programs, such as specialization, master's and PhD degrees; number of specialists working in the country, and professional practice (how a professional carries out his role or carries out his activities in his area of expertise).

When questionnaires returned, it was constructed a database with data from the participating countries. The SPSS (Statistical Package for Social Science for Windows, Inc., USA) version 22.0 for Windows® was used to perform the statistical analysis. The results were presented descriptively.

## Results

The questionnaires were returned from all 11 countries participating in the study. Table 1 shows the data on recognition of OP and OM, academic training and areas of practice by country. With the exception of Uruguay, all other countries have a recognized OP specialty. Brazil and Mexico were the first countries among those studied to recognize OP as a specialty in 1971 and 1975, respectively. On the other hand, Paraguay and Peru were the most recent to recognize OP as a specialty.

In the case of OM, from the countries studied, Uruguay and Bolivia do not recognize the specialty. In the case of Chile, OP and OM are recognized as a single specialty. Colombia and Brazil were the first countries to recognize OM as a specialty, respectively, in 1986 and 1992. The specialty is recognized by the professional regulatory bodies in each country that oversee the practice of the profession. In this case, the dental councils or boards. To obtain the title of specialist, you need to have completed a specialization or residency course recognized by the government and which has the minimum workload required, which varies from country to country. Regarding the existence and modalities of postgraduate programs, the most common was Master's degree programs in six countries (Argentina, Brazil, Mexico, Peru, Uruguay and Venezuela). On the other hand, the PhD level is offered by three countries, Argentina, Brazil and Mexico.

Residency programs in OP and OM are offered only in Brazil and Peru. The main field of action for the OP and OM specialties was in university teaching and in private clinics. Seven (63.63%) countries reported the work of professionals in hospitals. In Brazil, there are two important spaces for the work of OP and OM, which are the public health system and military forces. Ten countries have at least one professional society representing the specialties. Only Ecuador does not have a scientific society of the specialties studied.

Table 2 show population data, number of dentists, ratio of population and dentists by country and the number of OP and OM in each country.

The largest populations in the countries studied were from Brazil and Mexico, while Uruguay and Paraguay were the smallest. The ratio of dentists to the general population was quite varied. The lowest indicator was observed in Brazil (1:531.81) to the highest (1:3,255.61) in Ecuador. In addition to the OP and OM numbers in each country studied, we analyze the ratios between the number of OP and OM by the population of each country and in relation to the overall number of dentists. It can be seen that the numbers are quite varied. Paraguay has only one OP working in the country. Countries such as Peru and Venezuela have one OP for more than 4.5 million inhabitants. When looking at the number of OP in relation to dentists, the numbers also vary. Countries such as Paraguay and Peru have one OP for more than 9,000 dentists, while Mexico and Chile have one for approximately 440 and 269 dentists, respectively. In the case of OM, the numbers also vary greatly. Brazil, Mexico, Paraguay and Peru have only one OM for more than 1.8 million inhabitants. In the relationship between OM specialists and dentists, Paraguay and Peru have one professional for more than 3,000 dentists.

Another interesting variable (not shown in the Tables) refers to the number of undergraduate courses in dentistry. The countries with the most courses were Brazil (*n*=412) and Mexico (*n*=128), while those with the lowest number were Uruguay (*n*=2) and Venezuela (*n*=8).

## Discussion

This was the first study to specifically evaluated the current stage of OP and OM in the context of Latin America. Data from this study provide important information on the two specialties in the countries analyzed. Our study evaluated countries with very different populations and economic characteristics. An international multicenter study on specialized training and education in OP showed that training varies across the world. However, the authors feel there is sufficient commonality for the development of an agreed indicative framework on education and training in Oral and Maxillofacial Pathology (11). Although our study did not have the objective of directly evaluating the training received by OP and OM, it is clear that it would be very important to have better dialogue and build common actions between these Latin America countries.

A favorable factor is the presence of professional societies for both specialties (OP and OM) in all 11 countries except Ecuador. Scully *et al*., (2016) (2) evaluating OM across the globe: birth, growth and future, highlighted the importance of professional societies multinational, national or multistate and their performances. A second proposition of this study is to stimulate greater interaction between these professional societies through joint actions, as is sometimes observed in scientific events and multicenter studies in order to know and reduce the differences between OP and OM in the countries studied. Recently, we described the first 50 years of the history of the Brazilian Society of Oral Medicine and Oral Pathology (SOBEP) and it is observed that since its foundation, back in the seventies, of the last century, the SOBEP has expanded to over 300 active members. Annually, approximately 1,000 attendees meet in a national itinerant conference, which is held every July (3).

Another important result was to analyze the employment outcomes of the OP and OM. University teaching and private clinics were the most common work environments in all countries. The insertion in the hospital environment has also grown and this interaction with medicine in general is essential. Recently, we conducted a study with OP and OM and the findings suggest that Brazilian postgraduates in these fields have more opportunities for employment in private settings. Teaching/research were the most prevalent employment activity, despite being less than half of the sample (13).

With respect to continuing education programs, at the postgraduate level, of the eleven countries participating in the study, two do not have any type of postgraduate program (Ecuador and Paraguay). Bolivia had a single specialization class in Oral and Maxillofacial Pathology in 2013, which lasted two years. A new specialization course is currently underway, also lasting two years. Three countries offer postgraduate degrees at the PhD level (Argentina, Brazil and Mexico). Postgraduate, master's and specialization levels were the most common among the countries studied. In the present study, it was not possible to analyze the curricular structures of the postgraduate programs offered in each country, as well as the legal requirements in the regulatory agencies of each one. In Brazil, for example, there is the presence of a regulatory agency to control the supply of master's and PhD degrees (https://sucupira.capes.gov.br/sucupira/). The legal requirements are mainly related to the body of researchers and scientific production. Master's degrees have a duration of two years and PhDs of four years. Currently, in the country, there is only one Institution (FOP-UNICAMP) offering both levels of training in the areas of OP and OM (https://www.fop.unicamp.br/cpg/index.php/home-estomatopatologia-br). The primary objective of master's and PhD courses is to prepare individuals as educators and researchers in the field. The majority of graduates are equipped to practice, teach, and conduct research in OP and OM.

Residency programs offered in certain countries (Brazil and Peru) are postgraduate courses designed to provide professional training in the field, primarily emphasizing clinical practice. These programs are subject to government-affiliated regulatory agencies, and students are fully dedicated, receiving a scholarship for their participation. In contrast, specialization courses also emphasize clinical practice but with a lower workload than residency programs and are typically self-funded by individuals. Upon completion of either program, individuals are qualified to practice in the chosen field.

Another point of the present study would be to deepen the knowledge about the curricular structures of the graduate programs of the eleven countries evaluated and to think about common actions and successful experiences among them. In an international survey in postgraduate training in OM has been observed variation in OM training throughout the world. As curricula in postgraduate OM training are varied and quite detailed, it is difficult to capture their similarities and differences in one survey (14).

Another important dimension of the present study was the number of OP and OM in relation to the population of each country and in relation to the number of practicing dentists. The results were quite heterogeneous. In general, the number of OP was much lower than OM specialists. Countries such as Paraguay, Argentina, Peru and Venezuela have a very limited number of OP in relation to the population in general and, in the case of Paraguay in particular, a single professional. In OM the numbers also vary greatly, both in relation to the population of each country and in relation to dentists. Countries such as Brazil, Mexico, Paraguay and Peru have a very small number of OM for the general population. Although it is a limitation of the present study not to know the main reasons for these numbers, it is known that other dental specialties arouse greater interest in graduates, as well as the limitations as well as the limitations of insertion in the work environments.

Some limitations of this study were related to the impossibility of deepening some of the dimensions analyzed, for example, the reality of postgraduate programs and the distribution of OP and OM professionals in each country.

In summary, our study proposes a permanent collaboration between the countries of Latin America, sharing successful experiences in the fields of OP and OM and to develop joint actions for the limitations highlighted between the countries, such as continuing education in postgraduate programs (curricular structure), collaborative research projects, and the quantity that are training in OP and OM.

## Figures and Tables

**Table 1:** General characteristics of Oral Pathology (OP) and Oral Medicine (OM) specialties in Latin America.

Country	Recognition of OP	Year	Recognition of OM	Year	Postgraduate programs	Areas of activity	Professional society
Argentina	Yes	2006	Yes	2006	Specialization, Master's and PhD	Hospitals, Private Clinical and Universities in Teaching	Argentine Society of Oral Medicine
Bolivia	Yes	2007	No	-	No	Universities in Teaching and Private Clinical	Society of Bucco Maxillofacial Pathology Bolivia
Brazil	Yes	1971	Yes	1992	Specialization, Residency Program, Master's and PhD	Hospitals, Private Clinical, Universities in Teaching and Public Health System and Military Forces	Brazilian Society of Stomatology and Oral Pathology
Chile	Yes	2007	Yes	2007	Specialization	Hospitals, Private clinical and Universities in Teaching	Society of Bucomaxillofacial Pathology of Chile
Colombia	Yes	1992	Yes	1986	Specialization	Hospitals, Private clinical and Universities in Teaching	Colombian Academy of Oral Pathology and Colombian Association Of Stomatology, Semiology and Oral Surgery
Ecuador	Yes	-	Yes	-	No	Hospitals, Private clinical and Universities in Teaching	There is no Professional society
Mexico	Yes	1975	Yes	-	Specialization, Master's and PhD	Universities in Teaching, Private Clinical	Mexican Association of Pathology and Oral Medicine, Colegio A.C.
Paraguay	Yes	2023	Yes	2023	No	Universities in Teaching, and Private Clinical	Paraguayan Society of Oral Medicine and Pathology
Peru	Yes	2020	Yes	-	Residency Program, Specialization, and Master	Hospitals, Private Clinical, Universities in Teaching or Pathology Services	Peruvian Association of Oral and Maxillofacial Medicine and Pathology
Uruguay	No	-	No	-	Master	Hospitals, Private Clinical and Universities in Teaching	Uruguayan Society of Stomatological Pathology
Venezuela	Yes	1980	Yes	2005	Master	Hospitals, Private Clinical and Universities in Teaching	Venezuelan Society of Oral Medicine and Venezuelan Society of Oral Pathology

**Table 2:** Population data, number of dentists and specialists in Oral Pathology (OP) and Oral Medicine (OM) in Latin America.

Country	Population (Millions)	Number of dentists	Ratio of population to dentists	OP specialists	OM specialists
Argentina	48.8	63.500	721.25	Less than 100	Less than 10
Bolivia	11.2	15.000	747.73	10	0
Brazil	203	402.961	503.77	422	1,072
Chile	20	32.543	614.57	74	74
Colombia	52.2	60.000	870.25	More than 50	More than 100
Ecuador	16.9	5.203	3,255.61	Unregistered	Unregistered
Mexico	129	70.000	1,842.85	260	27
Paraguay	7.5	9.106	829.5	1	3
Peru	33.7	55.324	609.60	18	6
Uruguay	3.4	3.880	892.19	6	14
Venezuela	29	41.994	691.39	63	6
